# New approaches to studying early brain development in Down syndrome

**DOI:** 10.1111/dmcn.14260

**Published:** 2019-05-17

**Authors:** Ana A Baburamani, Prachi A Patkee, Tomoki Arichi, Mary A Rutherford

**Affiliations:** ^1^ Centre for the Developing Brain Department of Perinatal Imaging and Health School of Biomedical Engineering & Imaging Sciences King's College London King's Health Partners St Thomas’ Hospital London UK; ^2^ Department of Bioengineering Imperial College London London UK; ^3^ Children's Neurosciences Evelina London Children's Hospital London UK

## Abstract

Down syndrome is the most common genetic developmental disorder in humans and is caused by partial or complete triplication of human chromosome 21 (trisomy 21). It is a complex condition which results in multiple lifelong health problems, including varying degrees of intellectual disability and delays in speech, memory, and learning. As both length and quality of life are improving for individuals with Down syndrome, attention is now being directed to understanding and potentially treating the associated cognitive difficulties and their underlying biological substrates. These have included imaging and postmortem studies which have identified decreased regional brain volumes and histological anomalies that accompany early onset dementia. In addition, advances in genome‐wide analysis and Down syndrome mouse models are providing valuable insight into potential targets for intervention that could improve neurogenesis and long‐term cognition. As little is known about early brain development in human Down syndrome, we review recent advances in magnetic resonance imaging that allow non‐invasive visualization of brain macro‐ and microstructure, even in utero. It is hoped that together these advances may enable Down syndrome to become one of the first genetic disorders to be targeted by antenatal treatments designed to ‘normalize’ brain development.

**What this paper adds:**

- Magnetic resonance imaging can provide non‐invasive characterization of early brain development in Down syndrome.
- Down syndrome mouse models enable study of underlying pathology and potential intervention strategies.
- Potential therapies could modify brain structure and improve early cognitive levels.
- Down syndrome may be the first genetic disorder to have targeted therapies which alter antenatal brain development.

## What this paper adds

- Magnetic resonance imaging can provide non‐invasive characterization of early brain development in Down syndrome.
- Down syndrome mouse models enable study of underlying pathology and potential intervention strategies.
- Potential therapies could modify brain structure and improve early cognitive levels.
- Down syndrome may be the first genetic disorder to have targeted therapies which alter antenatal brain development.

This article's abstract has been translated into Spanish and Portuguese.

Follow the links from the abstract to view the translations.

Video Podcast: https://www.youtube.com/watch?v=x4x4iaP7ozk&feature=youtu.be

## Resumen

**Nuevos enfoques para estudiar el desarrollo cerebral temprano en el síndrome de Down:**

El síndrome de Down es el trastorno del desarrollo genético más común en los seres humanos y es causado por la triplicación parcial o completa del cromosoma 21 (trisomía 21). Es una condición compleja que se traduce en múltiples problemas de salud a lo largo de toda la vida, incluidos diversos grados de discapacidad intelectual y retrasos en el habla, la memoria y el aprendizaje. Debido a que la duración y la calidad de vida están mejorando para las personas con síndrome de Down, ahora se está prestando atención a la comprensión y al tratamiento de las dificultades cognitivas asociadas y sus sustratos biológicos subyacentes. Estos estudios han incluido estudios de imagen y postmortem que han identificado volúmenes cerebrales regionales disminuidos y anomalías histológicas que acompañan a la demencia de inicio temprano. Además, los avances en el análisis del genoma completo y los modelos de ratones con síndrome de Down brindan información valiosa sobre los posibles objetivos de la intervención que podrían mejorar la neurogénesis y la cognición a largo plazo. Como se sabe poco sobre el desarrollo temprano del cerebro en el síndrome de Down humano, revisamos los avances recientes en imágenes de resonancia magnética que permiten la visualización no invasiva de la macro y microestructura cerebral, incluso en el útero. Se espera que, en conjunto, estos avances puedan permitir que el síndrome de Down se convierta en uno de los primeros trastornos genéticos a los que se aplican tratamientos prenatales diseñados para “encauzar” el desarrollo cerebral.

## Resumo

**Novas abordagens para o estudo de desenvolvimento cerebral precoce na síndrome de Down:**

A síndrome de Down é a desordem desenvolvimental de origem genética mais comum em humanos. É causada por triplicação parcial ou completa do cromossomo 21 (trissomia do 21). Trata‐se de uma condição complexa que resulta em múltiplos problemas de saúde ao longo da vida, incluindo graus variados de deficiência intelectual, e atrasos na fala, memória e aprendizagem. Como tanto a duração quanto a qualidade de vida têm melhorado para indivíduos com síndrome de Down, agora a atenção se volta para compreender e potencialmente tratar dificuldades cognitivas associadas e seus substratos biológicos de base. Incluem‐se estudos de imagem e pós‐morte que identificaram menores volumes cerebrais e anomalias histológicas que acompanham a demência de início precoe. Além disso, avanços na análise do genoma em modelos de ratos com síndrome de Down fornecem informações valiosas sobre potenciais alvos para intervenção que podem melhorar a neurogênese e a cognição em longo prazo. Como pouco se sabe sobre o desenvolvimento cerebral precoce na síndrome de Down, nós revisamos avanços recentes em imagens por ressonância magnética que permitem visualização não‐invasiva da macro‐ e micro‐estrutura do cérebro, mesmo no útero. Espera‐se que, juntos, estes avanços possibilitem que a síndrome de Down se torne a primeira desorgem genética a ser alvo de tratamentos antenatais voltados para “normalizar” o desenvolvimento cerebral.

**Abbreviations**
CHD: Congenital heart defects
Hsa21: Human chromosome 21
Mmu: Mouse chromosome

Down syndrome is caused by partial or complete triplication of human chromosome 21 (Hsa21; trisomy 21) and is the most common genetic developmental disorder in humans. It is a complex condition which results in multiple lifelong health problems, including varying degrees of intellectual disability and delays in speech, memory, and learning. Worldwide, Down syndrome affects 1 in 1000 to 1100 live births annually. Whilst there have been significant improvements in non‐invasive prenatal screening,^1^ the prevalence of Down syndrome has remained relatively unchanged over the past 30 years, partly because of increasing maternal age.^2^ In addition to cognitive difficulties, there is typically multisystem involvement, with comorbidities including congenital heart defects (CHD; 40–50%), hypothyroidism, hearing, vision, and gastrointestinal complications. In early adulthood, cognitive decline is common with a high risk of early onset dementia and Alzheimer disease. In recent years, increased research, education, health care, and intervention programs have all contributed to people with Down syndrome now working and leading longer, healthier lives.

As Down syndrome is a multigene, multisystem disorder, accurately predicting neurocognitive abilities through the lifespan and understanding the high degree of variability across functional phenotypes remains a significant challenge. As a result, most clinical research in Down syndrome has been focused on understanding the pathogenesis of early onset dementia in adults, and clinical trials with pharmacological agents have been focused on improving cognition and delaying the development of dementia in adolescents and adults. Here, neuroimaging has become an increasingly useful modality to understand the progression of the underlying brain abnormalities and monitor the effects of potential therapeutic intervention. In contrast, published brain phenotypes during the fetal and neonatal period have been limited to only a handful of small postmortem case series. Therefore, whilst such studies have provided vital information about how early brain development is altered in Down syndrome, by nature, they cannot inform about the natural history of the abnormalities and crucially do not allow correlation of the identified brain phenotypes with subsequent outcome. In this review we describe how recent advances in developmental animal models of Down syndrome and non‐invasive imaging methods can fill this gap in knowledge by enabling the first in vivo studies in early human life.

## Individual Variability

Hsa21 contains 222 protein coding genes, and 325 non‐protein encoding genes.^3^ Studies of partial trisomy of Hsa21 have revealed that multiple regions of Hsa21 contribute to the observed physical and neurodevelopmental characteristics of Down syndrome.^4^, ^5^ The phenotype in Down syndrome is thought to arise from the overexpression and dysregulation of these genes and their associated pathways, together with global cellular stress responses and compensatory mechanisms early in development.^6^ Epigenetic changes have also been observed in the fetal brain and blood from newborn infants with Down syndrome^7^, ^8^ which may further impact development and contribute to the range of observed cognitive outcomes. The neurological phenotype constantly changes over the life span of Down syndrome,^9^ with differences continuing into adulthood. From 20 to 40 years of age, the majority of individuals with Down syndrome appear to develop characteristic Alzheimer disease neuropathology such as amyloid‐β plaques and neurofibrillary tangles; however, not all will develop dementia, which has a clinical prevalence of 68 to 80 per cent by 65 years of age.^9^, ^10^, ^11^, ^12^ Dementia is also strongly associated with early mortality in older (>36y) adults with Down syndrome.^13^ There are therefore multiple factors which may explain why individual differences exist across *all* levels of assessment: from gene expression, cellular responses, and subsequent brain development, to cognitive, motor, and behavioural phenotypes.^6^, ^12^, ^14^

## Neurodevelopment in Down Syndrome

Variable but atypical behavioural and cognitive functioning emerges throughout the lifespan in Down syndrome. IQ ranges from mild to severe disability (30–70; average IQ 50),^15^ with females reported to have milder degrees of intellectual disability compared to males.^15^, ^16^ Varying degrees of impairment in speech and language, memory, learning, and motor functions are also present.^9^, ^14^, ^17^, ^18^, ^19^

In comparison to typically developing controls, infants with Down syndrome may have only mild delays in learning and cognition during early infancy. Delayed or impaired cognitive and behavioural function then becomes more prominent from 2 years of age, with the rate of intellectual development slowing with increasing age.^12^, ^20^ Toddlers and young children with Down syndrome also have a higher prevalence (pooled prevalence of 16%) of autism spectrum disorder, which is further increased in those with greater cognitive impairment.^21^, ^22^, ^23^ Epilepsy (and in particular West syndrome) occurs at a higher incidence (1–13%) in children with Down syndrome.^24^, ^25^ Recent studies also suggest that preschool age children with an associated CHD (typically atrioventricular septal defects and ventricular septal defects) have poorer neurodevelopmental outcomes.^26^, ^27^, ^28^, ^29^ This wide spectrum of outcomes and limited understanding regarding how they relate to the underlying brain abnormalities therefore can significantly hamper antenatal parental counselling and undermine attempts to identify and assess potential treatment strategies.

### Neurological phenotype – what is known from postmortem and adult studies

It has been widely reported that children and adults with Down syndrome have smaller whole brain volumes, and a smoother, simplified gyral appearance.^30^ Reduced cortical surface area and increased cortical thickness have also been observed in children and young adults with Down syndrome.^31^ By middle adulthood, premature structural brain ageing can be detected.^32^ This includes disproportionate volume reduction of the brain regions crucial for speech, learning, and memory such as the prefrontal cortex, hippocampus, and cerebellum.^30^, ^33^

Despite their small number of cases, postmortem studies have provided important information about the range and high variability of the neuropathological features evident in Down syndrome across the life‐span. These studies suggest that the known reductions in brain size (2D and 3D measures) and weight emerge during the fetal and newborn period.^34^, ^35^ During the second trimester, reduced cellular proliferation and increased cell death reflect the observation that fewer neurons are seen in the neocortex, hippocampus, and cerebellum.^36^, ^37^, ^38^, ^39^, ^40^ Fewer neurons in the ventricular zone and subventricular zone further suggest an underproduction of excitatory neurons, leading to enhanced inhibitory neural activity that may underlie some of the cognitive deficits observed in Down syndrome.^37^, ^40^, ^41^ A reduction in serotonin levels has also been described in fetal brains with Down syndrome.^42^ In addition, there is growing evidence of a greater shift towards neural progenitor cells differentiating into glia (microglia, astrocytes, and oligodendrocytes),^37^, ^43^ resulting in altered regional expression and cellular densities of glia and macrophages across gestation.^44^ During late gestation, when neocortical expansion occurs, brains with Down syndrome also show delayed and disorganized patterns of cortical lamination.^45^, ^46^

After birth (and described up to 14y) there is a profound decrease in neuronal number (20–50%) and altered morphology of dendritic spines across the cortical layers.^35^, ^47^, ^48^ From 3 months of age, more distinct deviations in brain growth and shape become evident, these include shorter anterior–posterior diameter, flatter occipital poles, and smaller frontal lobes, cerebellum, and brainstem.^35^ These are accompanied by reductions in synaptic density and length, and fewer dendritic spines (that are thinner and shorter in length).^35^, ^48^, ^49^, ^50^ Delays in myelination are also observed postnatally (from 2mo), which correlates with poorer psychomotor development.^51^ Collectively, these observations are associated with overexpression of dosage‐sensitive genes including (but not exclusively): *DYRK1A*,* APP*,* S100*β, and *OLIG2*, all located on Hsa21.^43^, ^46^, ^52^, ^53^ In addition, Cu/Zn superoxide dismutase (also on Hsa21) is suggested to contribute to increased oxidative stress and mitochondrial dysfunction.^46^, ^54^

## Mouse Models of Down Syndrome

Advances in genome‐wide analysis and the development of animal models have provided valuable insight into understanding gene dosage imbalances in disorders such as Down syndrome.^55^ Mouse models of Down syndrome have been crucial to help investigate the genetic and developmental origins of the Down syndrome phenotype and importantly to test therapies that have the potential to improve neurogenesis and long‐term cognition.^56^, ^57^ Hsa21 shares synteny with a large proportion of mouse chromosome (Mmu) 16 (approximately 102 protein coding genes) and shorter regions of Mmu10 (37 protein coding genes) and Mmu17 (19 protein coding genes).^3^ These have all been key targets in generating mouse models of Down syndrome (for a comprehensive list of mouse models of Down syndrome see Herault et al.^57^). Importantly, as in human postmortem studies, an imbalance of excitatory and inhibitory neurons, impaired neurogenesis, synaptogenesis, and altered dendritic development are also observed in mouse models of Down syndrome (detailed reviews are available elsewhere).^9^, ^17^, ^18^, ^19^, ^41^, ^56^, ^58^, ^59^

The Ts65Dn mouse (B6EiC3Sn *a*/*A*‐Ts[17^16^]65Dn/J) has historically been very important in the study of Down syndrome as it is trisomic for 90 protein coding genes on Mmu16 (approximately 55% of orthologous genes to Hsa21). However, Ts65Dn mice contain an extra copy of 60 genes (35 protein coding) located on Mmu17 (orthologous to Hsa6) that are not triplicated in people with Down syndrome and the resultant Ts65Dn phenotypes may be more severe than those seen in the human condition or possess spurious phenotypes not relevant to Down syndrome.^3^, ^60^, ^61^ Other mouse strains have therefore been developed with partial trisomy of genes on Mmu16. The Ts1Cje strain (B6EiC3Sn‐Ts[16C‐tel]1Cje/DnJ) contains a partial trisomy of approximately 71 to 81 genes on Mmu16, but also monosomy of seven genes on Mmu12, and has a milder phenotype compared to Ts65Dn mice.^3^, ^62^ Early studies of partial human trisomies suggested that the Down syndrome phenotype was due to the increased gene dosage of a smaller number of specific genes, known as the Down syndrome critical region extending approximately 5.4Mb.^63^, ^64^ Using Cre‐LoxP technology, the Ts1Rhr mouse strain (B6.129S6‐Dp[16Cbr1‐Fam3b]1Rhr/J) replicates trisomy of the Down syndrome critical region (33 conserved and minimally conserved genes).^65^ However, additional studies into partial trisomies and advances in gene mapping strongly suggest that these genes alone are not sufficient to result in all Down syndrome phenotypes.^4^, ^5^, ^65^, ^66^

The Tc1 (B6129S‐Tc[HSA21]1TybEmcf/J) transchromosomic (trans‐species aneuploidy) mouse line contains a freely segregated copy of Hsa21. Although some chromosomal rearrangement and deletions have been identified in the construction process, it has allowed exploration of the relationship between specific Hsa21 genes (including those not found in the mouse) and phenotype.^3^, ^57^, ^67^ Whilst amyloid precursor protein (APP) is known to significantly contribute to the early‐onset of Alzheimer disease, recently it has been shown that triplication of other genes on Hsa21 (Tc1 mouse is 75% trisomic for Hsa21 genes) can exacerbate plaque formation and cognitive deficits in mice.^68^ Advances in chromosomal engineering have facilitated the design of more specific mouse models which include duplications of entire syntenic segments of Mmu16 (Dp[16]1Yey)/Dp16 (B6.129S7‐Dp[16Lipi‐Zbtb21]1Yey/J) and Dp1Tyb (Dp[16Lipi‐Zbtb21]1TybEmcf)], Mmu17 (Dp[17]1Yey), and Mmu10 (Dp[10]Yey). This has led to the development of the most complete ‘triple trisomic mouse’ which develops Down syndrome‐related neurological impairments.^69^ The Dp1Tyb and Dp16 contain the largest duplication of Mmu16, carrying an extra copy of 148 genes which is the entire region of Mmu16 that is orthologous to Hsa21 and does not perturb genes on any other chromosomes.^57^, ^69^, ^70^ Whilst the triple trisomic mouse is incredibly labour intensive and costly to produce, assessing each individual trisomic mouse is providing further insight into the contribution of gene imbalance to Down syndrome phenotype.

Studies in mouse models have primarily focused on understanding the pathology of the adult and ageing Down syndrome brain, despite knowledge that alterations in brain development are observed from fetal life. Comparison with human development can be challenging, as mice are postnatal brain developers with a gestational length of 19 to 21 days. The bulk of cortical neurogenesis occurs during the mouse embryonic period (corresponding to early fetal life in the human), but is ongoing into postnatal life in the hippocampus and cerebellum. The rate of cellular migration and maturation differ regionally, but around the time of (rodent) birth, postnatal day 1 to postnatal day 3, neural development is generally considered to be comparable to a preterm human infant of 23 to 32 weeks postmenstrual age. The brain growth spurt of rodents occurs at postnatal day 7 to postnatal day 10, which is comparable to a term human infant of 36 to 40 weeks postmenstrual age.^56^, ^71^ Alterations at both embryonic and postnatal ages have been reported in Ts65Dn and Ts1Cje mice^72^ and reviewed in several recent publications.^9^, ^41^, ^59^ More recently, the Dp16 strain did not show any forebrain defects embryonically (embryonic day 13.5–18.5), but did show delayed growth, and delayed acquisition of milestones postnatally and a decrease in cortical excitatory and interneuron populations were observed at postnatal day 15, but were not evaluated at earlier postnatal ages.^73^, ^74^ Comparisons of embryonic and adult gene expression, brain development, and mouse behaviour have recently been done in the Ts65Dn, Ts1Cje, and Dp16 mouse strains and suggest widespread differences between models.^74^ This highlights the importance of assessing which mouse models best mimic the human phenotype of interest and then choosing a mouse model that is best suited for studying a specific outcome or genotype/phenotype relationship.

## Current Therapeutic Approaches

Studies are being conducted in mouse models of Down syndrome to target dosage sensitive genes that are involved in defects and delays in neurogenesis and neurotransmission, oxidative stress, and neurodegeneration (a comprehensive list has been previously reviewed, see Gardiner et al.^18^ and Herault et al.^57^). Pharmacological treatments in mouse models with DYRK1a inhibitors, selective serotonin reuptake inhibitors (fluoxetine), and sonic hedgehog agonists during the prenatal and/or postnatal period have provided promising evidence of improved cellular and behavioural outcomes.^56^, ^57^ Whilst the vast majority of these studies have been done in Ts65Dn mice, they provide crucial proof‐of‐concept that cognition can be improved and that aspects of brain structure can be restored, even if the drugs are administered well after periods of neuronal migration and maturation have ceased.

Current clinical trials in adolescents and adults with Down syndrome are aimed at improving cognition and delaying progression into Alzheimer disease. Two groups of common medications used to treat the symptoms of Alzheimer disease, acetylcholinesterase inhibitors (Aricept/Donepezil, Rivastigmine) and N‐methyl‐D‐aspartate receptor antagonists (Memantine), are currently undergoing clinical trials in patients with Down syndrome.^56^, ^75^ With the ultimate aim of reducing amyloid toxicity and regulating myo‐inositol levels, s*cyllo*‐Inositol (ELND005) has recently also been shown to be well tolerated in a Phase II clinical trial in young adults with Down syndrome without dementia.^76^ In addition, further novel pharmacological interventions have also been developed based on the improved knowledge of the genes located on Hsa21 and their specific pathways, including DYRK1a inhibitors (Epigallocatechin gallate), a selective gamma‐Aminobutyric acid‐A α5 receptor negative allosteric modulator (Basmisanil/RG1662; CLEMATIS Study), and antioxidant vitamin E.^56^

### Searching for new therapeutic windows

Growing evidence suggests that alterations in key cellular processes result in permanent modifications in structure from a very early stage in brain development. It is therefore possible that an early life therapeutic window exists, during which atypical brain development could be potentially modified before the abnormalities and neurocognitive impairment are fully established. In current clinical practice, commonly used early interventions include physiotherapy, occupational therapy, and speech and language therapy which may help to improve the acquisition of developmental milestones in infants and children with Down syndrome in the absence of any known effective pharmacological intervention. Here it is important to consider that the identification of novel candidate agents is difficult, given the absence of detailed understanding of the very early neurobiological trajectory in Down syndrome.

## What We Do Not Know

There is a lack of understanding about when deviations in brain development arise in Down syndrome, how these relate to subsequent function, and whether they are further altered by additional congenital morbidities (e.g. cardiac defects). Such information is best monitored by in vivo studies that provide opportunities to follow development longitudinally.

### Comorbidities

CHD (without Down syndrome) are generally associated with impaired clinical neurodevelopment and an underlying reduction in cortical grey matter volumes, gyrification index (indicative of less complex cortical folding), and abnormal cortical microstructure in the neonatal period. These changes were further associated with reduced cerebral oxygen delivery.^77^, ^78^ This therefore highlights the importance of understanding the additional and as yet unexplored, effects of a CHD on brain development in Down syndrome. Mouse models are also useful for this as the Ts65Dn,^79^ Ts1Cje,^80^ Tc1,^81^ Dp1Tyb,^70^ and the genetically similar Dp16 mouse strains all develop CHD, which are identifiable by embryonic day 14.5. Importantly, Tc1 (38–55% based on background strain) and Dp1Tyb mice (61.5% of embryos) share many of the specific features of atrioventricular septal defects that are common in humans with Down syndrome.^70^, ^81^ Lana‐Elola et al.^70^ have elegantly generated a mouse mapping panel using segmented duplications ranging in size to identify the location of a 4.9Mb genomic critical region for CHD, which consists of 39 genes (two of which are required in triplication).

### Improve translation between human studies and mouse models

Two studies in both human and mouse models of Down syndrome have utilized transcriptomic analysis to characterize the specific gene networks and associated biological processes which are altered during prenatal and postnatal development.^6^, ^82^ The identification of consistently disturbed signalling pathways could aid the recognition of novel pharmacological treatments.^6^, ^82^ However, to translate the findings from bench to bedside, an improved understanding of how molecular alterations impact on neurobiological development is needed through: (1) better detailing of the human condition; and (2) cross‐species validation between Down syndrome mouse models and human Down syndrome.

## Advances in Fetal and Neonatal Magnetic Resonance Imaging

Whilst the aforementioned postmortem studies and animal models have provided significant insights into the neuropathology of Down syndrome, a true understanding of the natural history of the human condition and how the pathology relates to neurodevelopmental outcome is only possible through in vivo studies. Here, there is great potential for an enhanced understanding of in utero and neonatal brain development in Down syndrome through the application of recent advances in non‐invasive imaging. Although ultrasound provides valuable insights into gross fetal body and brain development, it cannot provide detailed information about region and tissue specific brain development and growth trajectories.

Therefore magnetic resonance imaging (MRI) is an attractive alternative which is safe, does not use ionizing radiation, and can provide more extensive, detailed biometric data across gestation including information about both brain macro‐ and microstructure.^83^, ^84^ Although there are MRI studies which have assessed structural brain volume in early childhood,^59^, ^85^ very little work has been done with infants younger than 2 years of age with Down syndrome. Quantitative early MRI data could be related to data derived from clinical, cognitive, and behavioural assessments, as well as genetic information, thus allowing a comprehensive understanding of the complex relationships which underpin the Down syndrome phenotype. Such studies are currently ongoing in adults with Down syndrome to assess the changes in brain structure and function associated with cognitive decline and progression into early onset Alzheimer disease.^86^

### Fetal MRI

**Figure 1:**
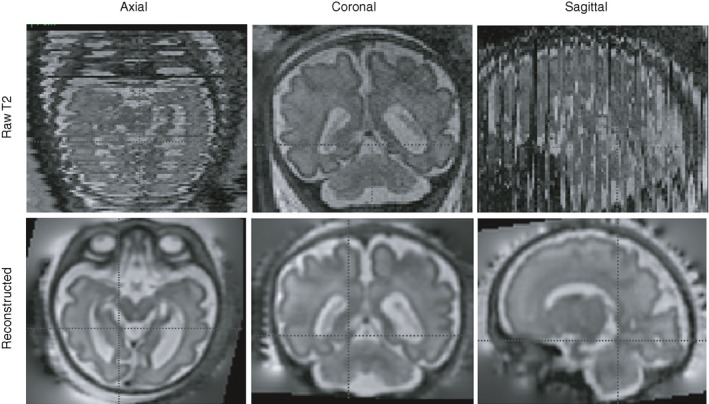
T2 fetal image reconstruction. Top row: One loop of single shot T2 images acquired in the coronal plane (centre). Numerous black lines in the sagittal and axial images represent missing or motion corrupted data. Bottom row: The reconstructed images have been obtained by registering several loops of single shot T2 images to provide high signal to noise, high resolution volumetric data sets.^87^, ^88^, ^89^, ^90^

Fetal MRI is challenging because of fetal motion, size, and position (relative to the surrounding maternal tissue). However, in comparison to ultrasound, it offers excellent soft tissue contrast and benefits from a wide field of view which allows the whole fetus to be imaged up until term gestation. To combat the effects of fetal and maternal motion, significant advances have been made in acquisition and processing protocols (such as optimized fetal motion correction and image registration pipelines) which can now provide high resolution and high signal to noise volumetric image data sets (Fig. 1).^87^, ^88^, ^89^, ^90^ This has now made it possible to obtain 3D magnetic resonance structural and functional data within 30 minutes of image acquisition using snapshot to volume reconstruction techniques.^88^, ^91^ These advances have led to increasing utilization of fetal MRI both in clinical practice and as a research tool to assess the fetal brain, heart, and organs, as well as the placenta.

### Neonatal MRI

Whilst imaging a newborn or young infant also presents technical and practical difficulties, there are now magnetic resonance compatible incubators and population‐specific processing pipelines to overcome these. Examples include a neonatal brain imaging system developed for the developing human connectome project (http://www.developingconnectome.org/) for non‐sedated sleeping infants, consisting of a neonatal head sized 32‐channel receive array coil and positioning system which significantly improves signal to noise ratio, an MRI safe trolley to minimize disturbance of the sleeping infant, additional ear protection, and a change in the start of magnetic resonance sequences to reduce the abrupt noise at the start of an acquisition sequence that may wake the infant.^92^

### Imaging infants and toddlers

During infancy and early childhood there is ongoing rapid growth of the cerebral cortex and maturation of white matter including myelination. Studying this population is therefore essential to provide a true characterization of these fundamental developmental processes and understand how a trajectory may deviate in pathological states. However, MRI of young children is associated with significant technical and practical challenges.^93^ As a result, clinically indicated magnetic resonance studies in children over 2 years of age are often done under general anaesthesia which would not be appropriate for research studies. Therefore in these children, other strategies have been explored to reduce anxiety, including mock‐scanner training sessions or a premeeting with the child and family to talk through the MRI process.^94^ Although children under 2 years of age may settle with oral sedation, this is less commonly done for research MRI scans because of increasing concerns about possible neurotoxicity.^95^ In this situation, coordinating with sleep, nap, or feeding times and modifying the magnetic resonance acquisition sequences to reduce sudden noise and/or volume may help avoid the use of sedation. Foam padding around the head and vacuum immobilization bags can also be used to reduce head motion.^96^ Although such approaches make scanning feasible, success rates are often variable, particularly for the sequences which provide quantitative magnetic resonance measures and are highly sensitive to motion artefact.

### Structural

**Figure 2:**
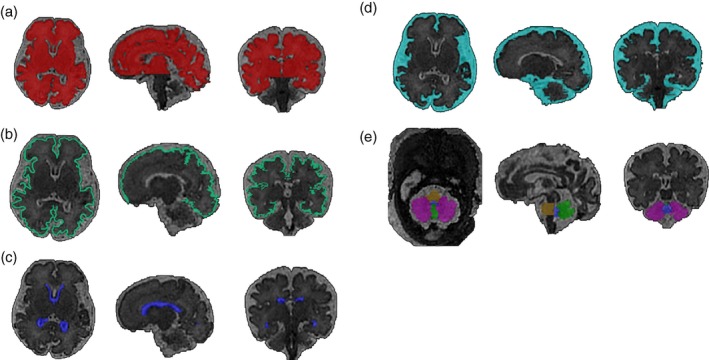
Segmentation of the brain from a fetus with Down syndrome at 33^+2^ gestational weeks. Semi‐automated segmentation of T2‐weighted volumetric magnetic resonance images showing (a) whole brain; excluding cerebellum (red), (b) cortex (green), (c) lateral ventricles (dark blue), (d) extra cerebral cerebrospinal fluid (light blue), (e) cerebellar hemispheres (purple), cerebellar vermis (bright green), pons (yellow), and fourth ventricle (blue).^97^ [Colour figure can be viewed at wileyonlinelibrary.com]

**Figure 3:**
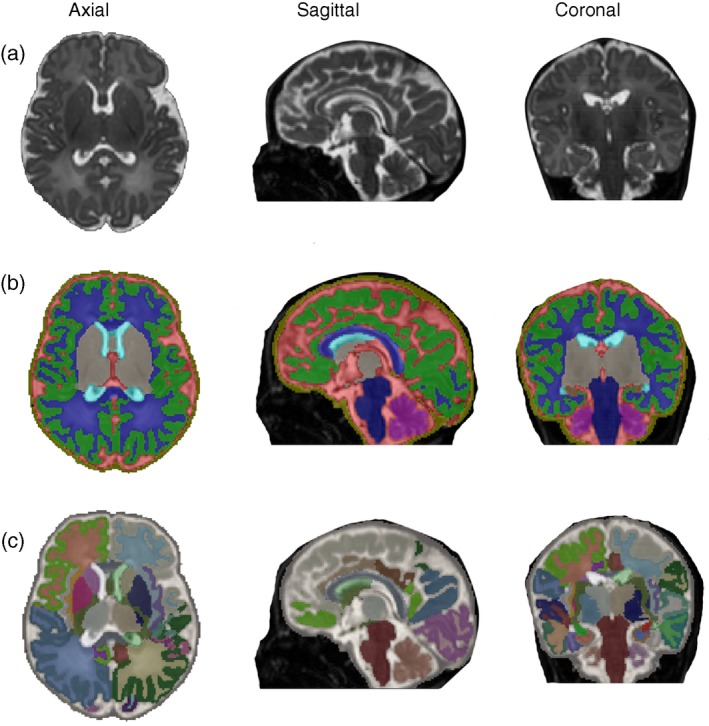
Automated segmentation of a brain from a neonate with Down syndrome at 42^+5^ weeks post menstrual age. T2‐weighted neonatal brain volumetric images in axial, sagittal, and coronal planes (left to right) segmented into multiple brain regions. (a) Raw T2 acquisition, (b) segmentation with nine regions of interest, and (c) segmentation with 87 regions of interest.^98^, ^99^, ^100^ [Colour figure can be viewed at wileyonlinelibrary.com]

**Figure 4:**
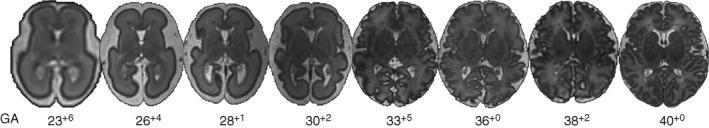
Fetal brain development. T2‐weighted axial images from fetal (23^+6^–38^+2^ GA) and neonatal (40^+0^ GA) magnetic resonance imaging showing development of the brain across gestation. Note the marked increase in cortical complexity with increasing gestation. GA, gestational age expressed as weeks+days.

**Figure 5:**
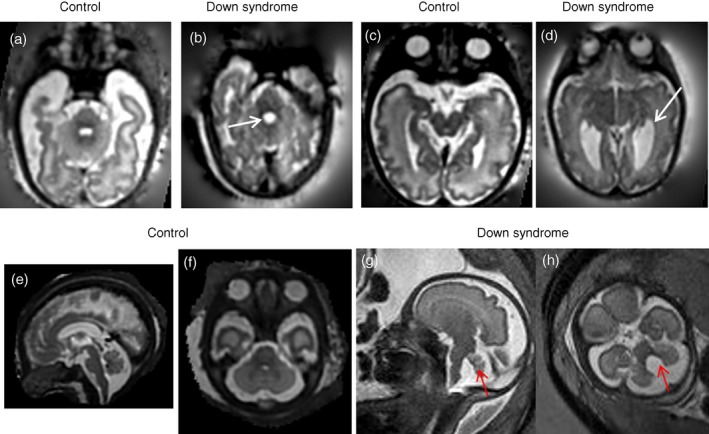
T2‐weighted fetal magnetic resonance imaging in a control fetus and a fetus with Down syndrome. T2‐weighted axial images showing the fourth (a,b) and lateral ventricle (c,d) in control (34^+1^ GA; a,c) and fetus with Down syndrome (33^+2^ GA; b,d). White arrows indicate enlarged fourth and lateral ventricles in a fetus with Down syndrome. T2‐weighted sagittal (e,g) and axial (f,h) images in a fetus with Down syndrome (30wks GA, g,h) compared to an age matched control (30wks GA, e,f). Red arrow indicates cerebellar vermis rotation (g) and fourth ventricle enlargement (h). GA, gestational age expressed as weeks+days. [Colour figure can be viewed at wileyonlinelibrary.com]

Single shot T1‐weighted and T2‐weighted sequences are conventionally used to visualize the structure and composition of the fetal brain and can provide regional 2D measurements and 3D volumetric information. The recent development of detailed atlases of the fetal^97^ (Fig. 2) and neonatal^98^, ^99^, ^100^ (Fig. 3) brain now allow robust automated or semi‐automated segmentation of brain regions and precise delineation of cortical sulcal and gyral development. This allows characterization of the normal trajectories of fetal brain growth and creation of population centile charts (available for 21–38wks gestation at: https://www.developingbrain.co.uk/fetalcentiles/; Fig. 4).^101^ Comparison with these typically developing growth charts therefore provides an ideal approach with which to assess, quantify, and identify when the deviations in regional and whole brain volumes seen in postmortem and adult Down syndrome brains are established. Our preliminary findings from fetal MRI scans show enlargement of the fourth and lateral ventricles, as well as cerebellar vermis rotation in a fetus with Down syndrome (Fig. 5).

### Diffusion MRI

**Figure 6:**
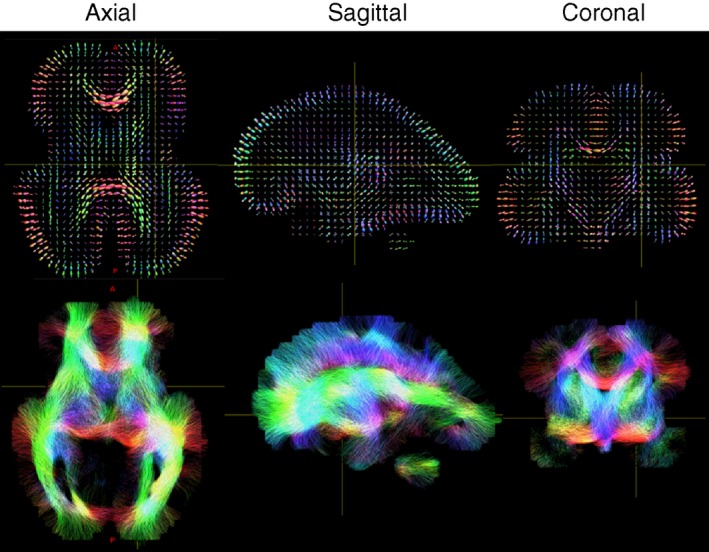
Fetal diffusion tensor imaging. Top row: Fibre orientations distributions per voxel. Bottom row: Tractography demonstrating major connections within the developing brain. [Colour figure can be viewed at wileyonlinelibrary.com]

Diffusion MRI can provide quantitative information about tissue microstructure and structural connectivity by measuring the total and directional diffusion of water molecules (Fig. 6). This can then provide specific measures which reflect white matter and cortical microstructure, such as fractional anisotropy which in high risk neonates significantly relates to later specific clinical neurodevelopmental impairment^102^, ^103^ and delays in cortical microstructural development.^104^ More complex models of voxel‐wise diffusion such as a fixel‐based analysis (fixel refers to a fibre population in a given voxel) can also provide measures of white matter fibre density, fibre cross‐sectional area, and the fibre orientation distribution.^105^ Other techniques, such as the neurite orientation distribution and density imaging model can also provide measures of the neurite density index and orientation dispersion index which may help explore the cortical synaptic and dendritic developmental abnormalities that are widely reported in Down syndrome.^9^, ^41^

Although diffusion MRI has not yet been used to study white matter and cortical microstructure in fetuses and neonates with Down syndrome, regional reductions in white matter microstructural integrity have been seen in adults with Down syndrome, which are more severe in those with additional signs of dementia.^106^ It has also been recently reported that children with Down syndrome (aged 2–4y) have reduced fractional anisotropy in supratentorial white matter tracts^107^ which mirrors both the reported delays in myelination in early childhood^51^ and transcriptome studies in both Down syndrome human brain (fetal) and Down syndrome mouse models (embryonic) studies which describe defective oligodendrocyte differentiation and myelination.^82^

### Functional MRI

Functional MRI provides an indirect measure of neural activity by detecting dynamic variation of the blood oxygen level‐dependant contrast caused by locally coupled changes in cerebral blood flow and haemoglobin oxygenation.^108^ This allows detailed mapping of functional activity which can be used to characterize the whole brain's large‐scale functional architecture. Studies in the neonate and more recently the fetus suggest that the perinatal period is of particular importance for the establishment of this architecture, as patterns of functional activity appear to rapidly increase in spatial complexity during this time.^109^, ^110^, ^111^, ^112^

In addition to analysing blood oxygen level‐dependant signal changes when an individual is presented a specific stimulus or performs a task (known as task‐based functional MRI), data can also be collected and analysed when an individual is at rest (known as resting state functional MRI). The latter can be used to identify spatial patterns of temporal correlation of intrinsic signal fluctuations (known as functional connectivity). Altered patterns of functional connectivity are seen in neuropsychiatric conditions and therefore may provide a suitable biomarker for abnormal brain function and predicting later adverse neurodevelopment in Down syndrome.^113^ In keeping with this, impaired functional connectivity and a simplified network architecture has been described in adolescents and young adults with Down syndrome.^114^ Functional connectivity could therefore potentially be used as a biomarker to monitor the outcome of clinical trials, as in a recent Phase II clinical trial in young adults with Down syndrome.^115^ By combining diffusion MRI and functional MRI data, there is the potential to provide further insights into the complex relationship between the brain's structural connections and its activity patterns, and crucially how it is altered by different pathological states.^116^

### Magnetic resonance spectroscopy

Magnetic resonance spectroscopy can non‐invasively quantify biochemical composition by sampling the resonant signal generated by hydrogen protons (^1^H) (and less commonly other nuclei with an odd mass number [sodium ^23^Na or phosphorus ^31^P]) from a voxel placed on a specific region of interest. Proton magnetic resonance spectroscopy is most commonly used in human brain studies because of the abundance of hydrogen in human tissue. The resultant spectra demonstrate metabolite peaks at a specific frequency (parts per million). Specific brain metabolites that can be quantified include myo‐inositol (osmoregulation, glial cell marker), choline (cell membrane), creatine (energy metabolism), N‐acetyl aspartate (neuronal marker and/or marker for mitochondrial function), and lactate (anaerobic glycolysis).^117^ The levels of different metabolites are often expressed as ratios rather than absolute metabolite quantification, particularly in cases with pathology where the detected changes may be subtle. Such measurements are of particular relevance in fetal and neonatal life as ongoing processes such as neuronal and glial proliferation, differentiation, and maturation are associated with constant fluctuations in the levels of brain metabolites which can be measured using magnetic resonance spectroscopy (e.g. increases in N‐acetyl aspartate and decreases in measurable choline with increasing brain maturation).^117^ Of interest, increased brain myo‐inositol has been reported both within the basal ganglia of children with Down syndrome^118^ and in the hippocampus, occipital, and parietal regions in adults with Down syndrome.^119^, ^120^, ^121^ The correlation of altered brain metabolite levels, (such as N‐acetyl aspartate and N‐acetyl aspartate/myo‐inositol ratio) with cognitive function can also provide insight into the progression of dementia for adults with Down syndrome.^122^

## Future Directions

Although current cognitive interventions target children and adults with Down syndrome, evidence suggests that deviations in brain development begin early in fetal life. However, to understand how to potentially intervene at this earlier time point, we need far greater knowledge about how the Down syndrome brain grows and develops, what causes the variability of neurodevelopmental outcomes, and the genotypic/phenotypic relationship that occurs in Down syndrome.

Significant advances in fetal and neonatal MRI sequence acquisition, motion correction techniques, and analysis methods now allow detailed characterization of the spectrum of early imaging phenotypes.^84^ These essential developments are of both research and clinical importance. Such prognostic information can improve care, help to counsel parents, and could potentially identify new therapeutic windows for intervention early in development.

**Figure 7:**
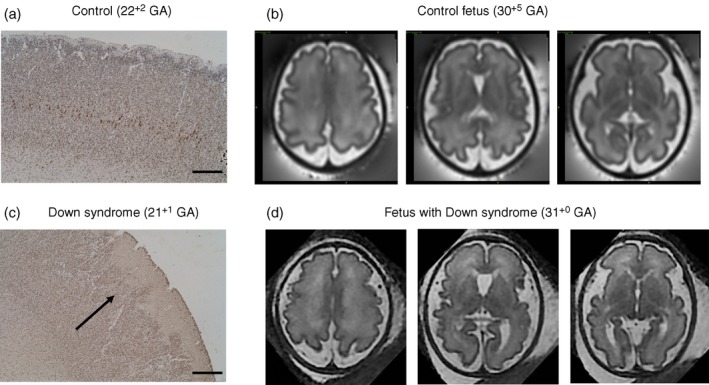
Neuronal staining in the cortex of human fetal postmortem tissue. HuC/HuD, a marker for all neurons in brain from control fetus at 22^+2^ GA (a) and fetus with Down syndrome at 21^+1^ GA (c). In the fetal brain with Down syndrome (c,d), the black arrow indicates evidence of aberrant cortical folding, a ‘wavy’ pattern which is in contrast to the control brain (a,b) (Research Ethics Committee UK: 07/H0707/139). Scale bar=500μm. T2‐weighted fetal magnetic resonance imaging in the axial plane show decreased cortical folding in a fetus with Down syndrome (d), compared to an aged matched control (b). GA, gestational age expressed as weeks+days. [Colour figure can be viewed at wileyonlinelibrary.com]

In addition, histological studies of human Down syndrome tissue at equivalent gestational ages can be used to determine the underlying neurobiological substrate for imaging phenotypes identified in the early developing brain in Down syndrome (Fig. 7). This combined early human data can be compared with that from available mouse models to identify those which most closely mimic the human condition and would therefore be suitable for use in interventional trials of early treatments designed to ‘normalize’ brain development and improve cognition.

The combination of preclinical animal, human postmortem, and in vivo imaging methods can therefore provide comprehensive and vital new insights into aberrant brain development in Down syndrome. This also has the potential to provide non‐invasive imaging based surrogate markers to predict later neurodevelopmental outcome. In the future, this novel early human imaging data can also be used in clinical trials as biomarkers to monitor the effectiveness of new therapies intervening during antenatal or neonatal time‐points.
